# Ex vivo detection of mandibular incisors’ root canal morphology using cone-beam computed tomography with 4 different voxel sizes and micro-computed tomography

**DOI:** 10.1186/s12903-023-03376-2

**Published:** 2023-09-09

**Authors:** Bingbing Bai, Ying Tang, Yihan Wu, Fan Pei, Qi Zhu, Peng Zhu, Yongchun Gu

**Affiliations:** 1 The Stomatology Hospital Affiliated of Suzhou Vocational Health College, Renmin Road 829#, Gusu Dist, Suzhou, 215002 China; 2 Department of Central Laboratory and Dentistry, Ninth People’s Hospital of Suzhou, Soochow University, Ludang Road 2666#, Wujiang Dist, Suzhou, 215200 China

**Keywords:** Cone-beam computed tomography, Voxel size, Mandibular incisor, Double root canal system, Accessory canal, Micro-computed tomography

## Abstract

**Background:**

In recent years, cone-beam computed tomography (CBCT) has been widely used to evaluate patients’ root canal anatomy due to its high resolution and noninvasive nature. As voxel size is one of the most important parameters affecting CBCT image quality, the current study evaluated the diagnostic potential of CBCT with 4 different voxel sizes in the detection of double root canal systems and accessory canals (ACs) in permanent mandibular incisors.

**Methods:**

A total of 106 extracted mandibular permanent incisors were collected from the dental clinics, and then were scanned by using micro-CT with a voxel size of 9 μm. The teeth were then fixed in the tooth sockets of human dry mandibles and scanned by using a CBCT device with 4 different voxel sizes (300, 200, 250, and 125 μm). Four observers detected in blind the root canal morphology of the teeth according to the CBCT images, and the presence or absence of a double root canal system, and the presence or absence of ACs, were scored according to a 5-point scale, respectively. Receiver operating characteristic (ROC) analysis was performed, and DeLong test was used to compare the area under the curve (AUC) values and the micro-CT data was taken as a gold standard.

**Results:**

Among 106 sample teeth, 25 specimens with a double root canal system were identified by the micro-CT. ROC curve analysis of the data obtained by the four observers showed that in the detection of double root canal systems, the AUC values ranged from 0.765 to 0.889 for 300 μm voxel size, from 0.877 to 0.926 for 250 μm voxel size, from 0.893 to 0.967 for 200 μm voxel size, and from 0.914 to 0.967 for 125 μm voxel size (all *p* < 0.01). In general, we observed a trend that the AUC values, sensitivity, and specialty increased with the decrease in the voxel size, and significantly higher AUC values were detected in 125 μm voxel size images. In the detection of ACs, ROC curve analysis showed that among the four observers, the AUC values ranged from 0.554 to 0.639 for 300 μm voxel size, from 0.532 to 0.654 for 250 μm voxel size, from 0.567 to 0.626 for 200 μm voxel size, and from 0.638 to 0.678 for 125 μm voxel size. CBCT images at a voxel size of 125 μm had a weak diagnostic potential (AUC: 0.5–0.7, all *p* < 0.05) in the detection of AC, with a lower sensitivity ranging from 36.8 to 57.9% and a higher specialty ranging from 73.6 to 98.8%.

**Conclusions:**

CBCT with 300 μm voxel size could only provide moderate diagnostic accuracy in the detection of a double canal system in mandibular incisors. CBCT with a voxel size of 125 μm exhibited high diagnostic value in the detection of double canal systems, while showing low but statistically significant value in the detection of ACs.

## Background

An exact knowledge of the individual root canal anatomy and variations is a prerequisite for successful endodontic treatment [1]. The mandibular central and lateral incisors are very similar in morphology and they usually have a single root and a single canal [2]. However, the presence of two canals in the bucco-lingual plane has been reported in mandibular incisors with detection rates ranging widely from 11 to 45%, and the discrepancy can be due to diverse laboratory and clinical methodologies used and different ethnicities of the population groups [3–15]. The second canal is often tiny in size and prone to be overlooked on conventional periapical radiographs during endodontic treatment, as they are 2-dimensional and cannot provide anatomic information of the root at the sagittal plane. Moreover, the image quality may be degraded due to distortion or superimposition of bone and other dental structures [10]. Failure to detect or treat an existing second canal may lead to unfavorable outcomes of endodontic treatment. The presence of accessory canals (ACs) is another endodontic challenge. They connect the main root canal or pulp chamber with the root surface and the incidence varies widely among different tooth types, and different populations [16]. ACs can result in lateral lesions or post-treatment periapical periodontitis as the conventional techniques are inadequate to access, clean, disinfect, and fill them efficiently [16]. Ji et al. [17] examined CBCT images of 70 extracted mandibular incisors taken at a voxel size of 125, 200 and 250 μm, and the detection accuracy of ACs were 80.0%, 13.3%, and 33.3% respectively (the clearing technique served as the gold standard). This finding indicated that assessment of ACs could only be performed with a voxel size of 125 μm (high-resolution scan mode). Due to the small size of ACs, previous studies were mostly based on laboratory methodologies such as micro-CT or clearing technique, and till now, no efficient diagnostic tool has been reported to clinically detect and identify ACs.

In recent years, dental cone-beam computed tomography (CBCT) has been widely used to evaluate the patients’ tooth root and canal anatomy due to its high resolution and noninvasive nature. Although the scan time and radiation dose of CBCT are significantly reduced as compared with conventional medical CT scans, CBCT scanners can still produce a higher radiation dose than a single traditional dental radiograph [18]. Therefore, the principles of ALARA (as low as reasonably achievable) and ALADA (as low as diagnostically acceptable) have to be followed, and no routine use of CBCT is allowed during dental treatments. The clinicians should be aware that CBCT imaging should be performed with the least amount of radiation required to provide adequate image quality, namely “dose optimization” [19]. The diagnostic accuracy of CBCT examination can be affected by several objective and subjective factors. The former includes the scanning unit itself, the field of view (FOV), scanning time, tube voltage and current, the voxel size, etc. [20, 21], while the latter includes but is not limited to the professional experiences and educational background of the observers. Voxel size is one of the most important parameters affecting the CBCT image quality. Generally speaking, a small voxel size in a CBCT protocol is often associated with better image quality and higher diagnostic accuracy [22, 23], but requires more exposure time and consequently may increase the radiation exposure to the patient [24]. Previous scholars have evaluated the impact of voxel size on different diagnostic tasks, such as detection of root fractures, carious lesions, root resorption, periodontal defects, erosions in the temporomandibular joint, root canal variations, etc., and the results varied depending on the task and the CBCT systems used [21, 25–28]. In regard to the diagnosis of root canal anatomy, Bauman et al. [22] and Mouzinho-Machado et al. [25] reported that a smaller voxel size increased the diagnostic accuracy of MB2 canals. While Vizzotto et al. [23] reported that filling of the first mesio-buccal canal (MB1) reduced the second mesio-buccal canal (MB2) detection in the CBCT images of 300 and 250 μm voxel size, but not of 200 μm voxel size; for teeth with MB1 filling, the 200 μm voxel size was the most appropriate, whereas for those with no MB1 filling or after MB1 root canal filling removal, the 300 μm voxel size was adequate to achieve good diagnostic performance and lower radiation exposure.

Micro-computed tomography (micro-CT) is another type of CT, which is often used for the ex vivo study of human teeth. Its resolution can be approximately 10-fold higher than that of CBCT, and thus it can be regarded as the gold standard for the diagnosis of root canal variations [16].

The aim of this study was to evaluate the ability of CBCT with 4 different voxel sizes in the identification of double root canal systems and ACs in permanent mandibular incisors, and the micro-CT images were used as the gold standard.

## Materials and methods

### Collection of sample teeth

Ethnic approval of the use of seven dry human mandibles and extracted human teeth was obtained from the Ethics Committee and the institutional review board (Issuing Number: KY2022-089-01) of the Ninth People’s Hospital of Suzhou. A total of 106 extracted permanent mandibular incisors were randomly collected from a native Chinese population as described previously [29], and the mean age of the subjects was 56.3 ± 16.7 years, ranging from 14 to 84 years.

### Micro-CT scanning and analysis

**Fig. 1:**
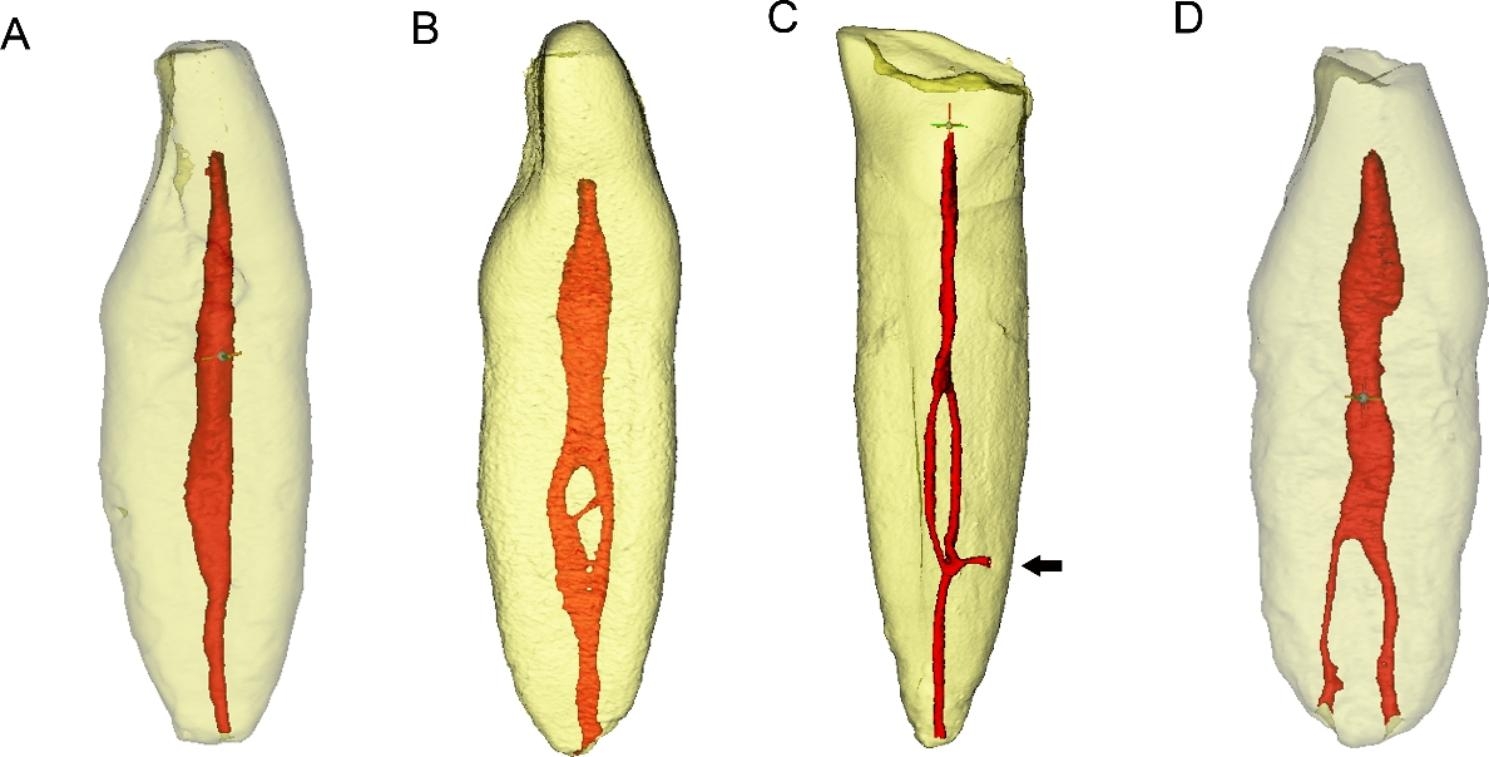
Representative 3D micro-CT images of root canal systems in mandibular incisors. **a** Absence of a double canal system (type 1–1). **b** Presence of a double canal system (type 1-2-1). **c** Presence of a double canal system (type 1-2-1), and presence of a lateral accessory canal (arrow). **d** Presence of a double canal system (type 1–2)

Each mandibular incisor was scanned by using a micro-CT scanner (SkyScan1174; Bruker-microCT, Kontich, Belgium) with a 1-mm aluminum filter at a voxel size of 9 μm, 800 mA, 50 kVp, rotation step of 0.7˚ and arch rotation of 180˚. The data sets were then transferred to Mimics 21.0 (Materialise, Leuven, Belgium) software to perform 3D reconstruction of the teeth and root canal systems. The presence or absence of a double root canal system and the presence or absence of AC(s) (lateral canals and/or apical accessory canals) (Fig. 1) were determined by the first author (*Bingbing Bai*), a junior dentist with working experience of 4 years.

Before micro-CT diagnosis, the calibration was performed by an endodontic specialist (*Yongchun Gu*) and the first author. The micro-CT images of 10 single- and 10 double-canaled incisors were randomly selected and examined at different views and magnifications to identify the presence or absence of double canals and ACs. Any disagreements were discussed until a consensus was reached. Cohen’s kappa test was used to evaluate the inter- and intra-observer errors. The micro-CT images were examined twice with an interval of 14 days. The intra-and inter-observer kappa values were both 1.0 (all *p* = 0.000).

The diameters of the ACs were measured by using Mimics software. Briefly, a cross-section was made at the midpoint of the accessory canal, and after full amplification of the image by zooming, the maximum (*D*) and minimum (*d*) diameters were measured.

### CBCT scanning and analysis

**Fig. 2:**
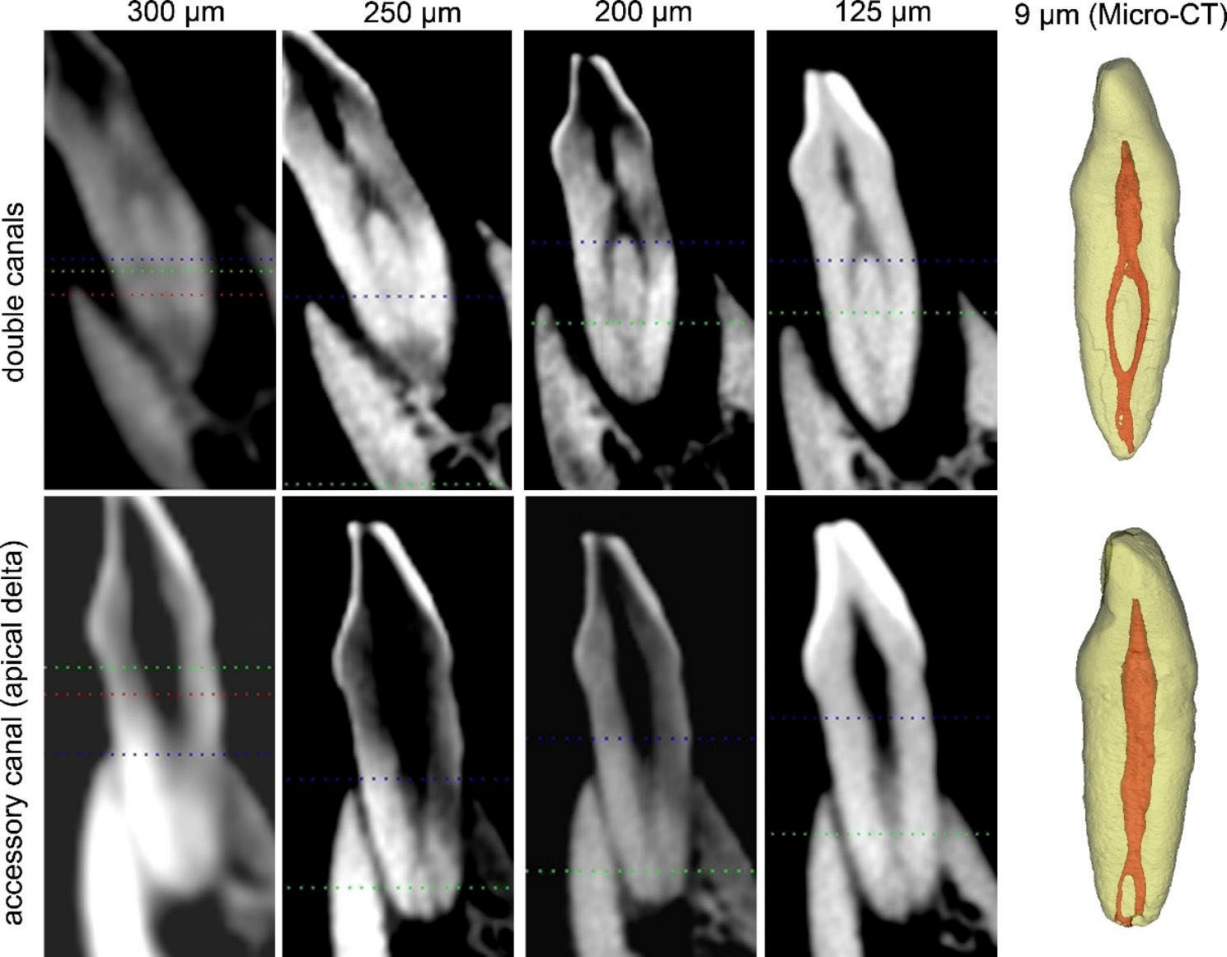
Representative CBCT (sagittal) and micro-CT (3D) images of mandibular incisors containing a double canal system (type 1-2-1) (upper-row figures) and an apical accessory canal (lower-row figures)

The extracted sample teeth were placed in the tooth sockets of the mandibular incisors in the dry mandible specimens and fixed with wax. Imaging was performed using a 3D eXam i (KaVo Co., Germany) CBCT machine with 4 different voxel sizes (Fig. 2). The imaging protocols were used in our clinical routine and listed as follows: (1) FOV = 14 × 8.5 cm; tube peak potential = 120 kVp; tube current = 15.44 mA; exposure time = 3 s; voxel size = 0.30 mm. (2) FOV = 14 × 8.5 cm; tube peak potential = 120 kVp; tube current = 30.89 mA; time = 6 s; voxel size = 0.25 mm. (3) FOV = 14 × 8.5 cm; tube peak potential = 120 kVp; tube current = 30.89 mA; time = 6 s; voxel size = 0.20 mm. (4) FOV = 8.5 × 8.5 cm; tube peak potential = 120 kVp; tube current = 30.89 mA; time = 6 s; voxel size = 0.125 mm.

Kavo eXam Vision software (KAVO, Germany) was used to analyze the images. The root canal anatomy was examined in detail by switching the viewing angle (horizontal plane, coronal plane, sagittal plane, and any other arbitrary planes) and adjusting the magnification. A blind visual analysis was conducted by 4 trained observers (a lab researcher with 9 years of experience working in the field of radiology [*Ying Tang*], an endodontic specialist with 25 years of working experience [*Yongchun Gu*], and 2 postgraduate students in endodontics [*Yihan Wu* and *Fan Pei*) who evaluated the CBCT images independently and scored the detection of a double root canal system on a 5-point rank scale: 1, definitely present of a double canal system; 2, probably present of a double canal system; 3, unsure; 4, probably absent of a double canal system; and 5, definitely absent of a double canal system (a single canal was definitely detected). The detection of ACs was also based on a 5-point rank scale: 1, AC definitely present; 2, AC probably present; 3, unsure; 4, AC probably absent; and 5, AC definitely absent. The observations of CBCT image sets with different voxel sizes were carried out according to the following sequence: 300 μm ◊ 250 μm ◊ 200 μm ◊ 125 μm. The interval between the two observations was 14 days to reduce the recall bias.

### Statistical analysis

Statistical analysis was performed using MedCalc statistical software version 15.8 (MedCalc Software bvba, Ostend, Belgium). Receiver operating characteristic (ROC) curve analysis was plotted to evaluate CBCT diagnostic accuracy with 4 different voxel sizes, and DeLong test was used to compare area under curve (AUC) values, and the observation results of micro-CT were taken as the gold standard. According to arbitrary guidelines, the accuracy of prediction is defined as low (AUC:0.5–0.7), moderate (0.7–0.9), and high (0.9-1). The sensitivity, specificity, positive predictive value, negative predictive value, and *Youden* index were calculated. The significance level was set at *p* < 0.05.

## Results

### The presence/absence of double canal systems or ACs in mandibular incisors detected by micro-CT

The root canal systems in mandibular incisors detected by micro-CT (the gold standard) were described in Table 1; Fig. 1. Among a total of 106 specimens, double root canal systems were detected in 25 specimens, and 26 ACs were identified in 19 specimens. The 25 cases of double canal systems included 21 cases of Vertucci’s type III (1-2-1) canals, 1 case of type II (2 − 1), and 3 cases of type V (1–2)] canals, and in the current study, we have not detected other complex root canal forms.

**Table 1:** Root canal configuration of mandibular incisors detected by micro-CT (*n* = 106)

	Double canals			Accessory canals	
	Presence	Absence		Presence	Absence
*n*	25	81		19	87
%	23.58%	76.42%		17.92%	82.08%

### The diameter of the ACs measured by micro-CT

**Fig. 3:**
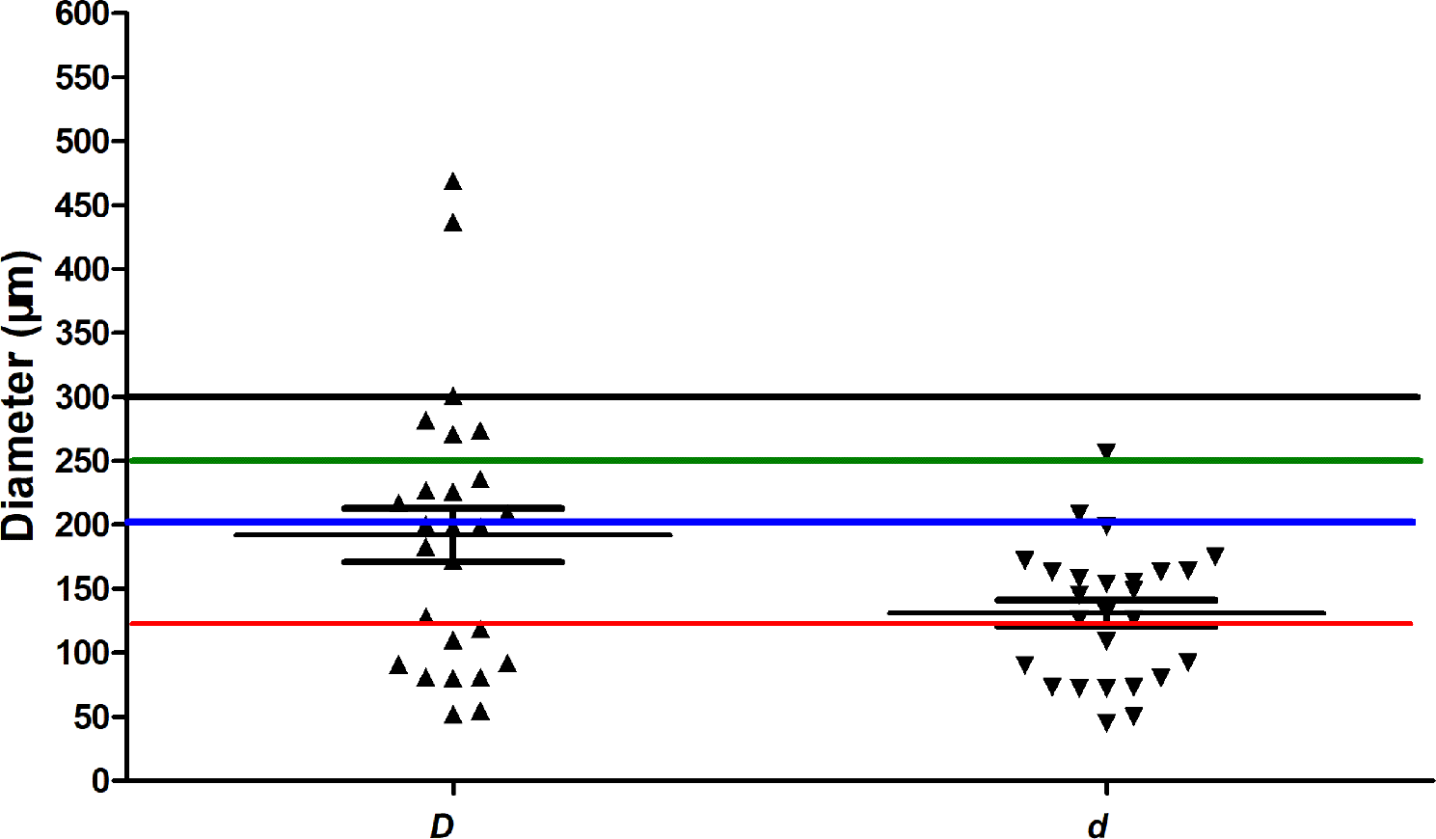
Micro-CT measurement results of maximum and minimum diameters of the accessory canals in mandibular incisors. Error bar is SEM

In 19 specimens, 26 accessory canals were detected by micro-CT scans. Measurement results of the maximum and minimum diameters of the ACs were shown in Fig. 3. The mean maximum and minimum diameter was 192 ± 107 μm and 131 ± 51 μm, respectively. The majority of the ACs (20/26) have a canal diameter (*D*) below 250 μm.

### The presence/absence of a double canal system in mandibular incisors detected by CBCT with 4 different voxel sizes

**Fig. 4:**
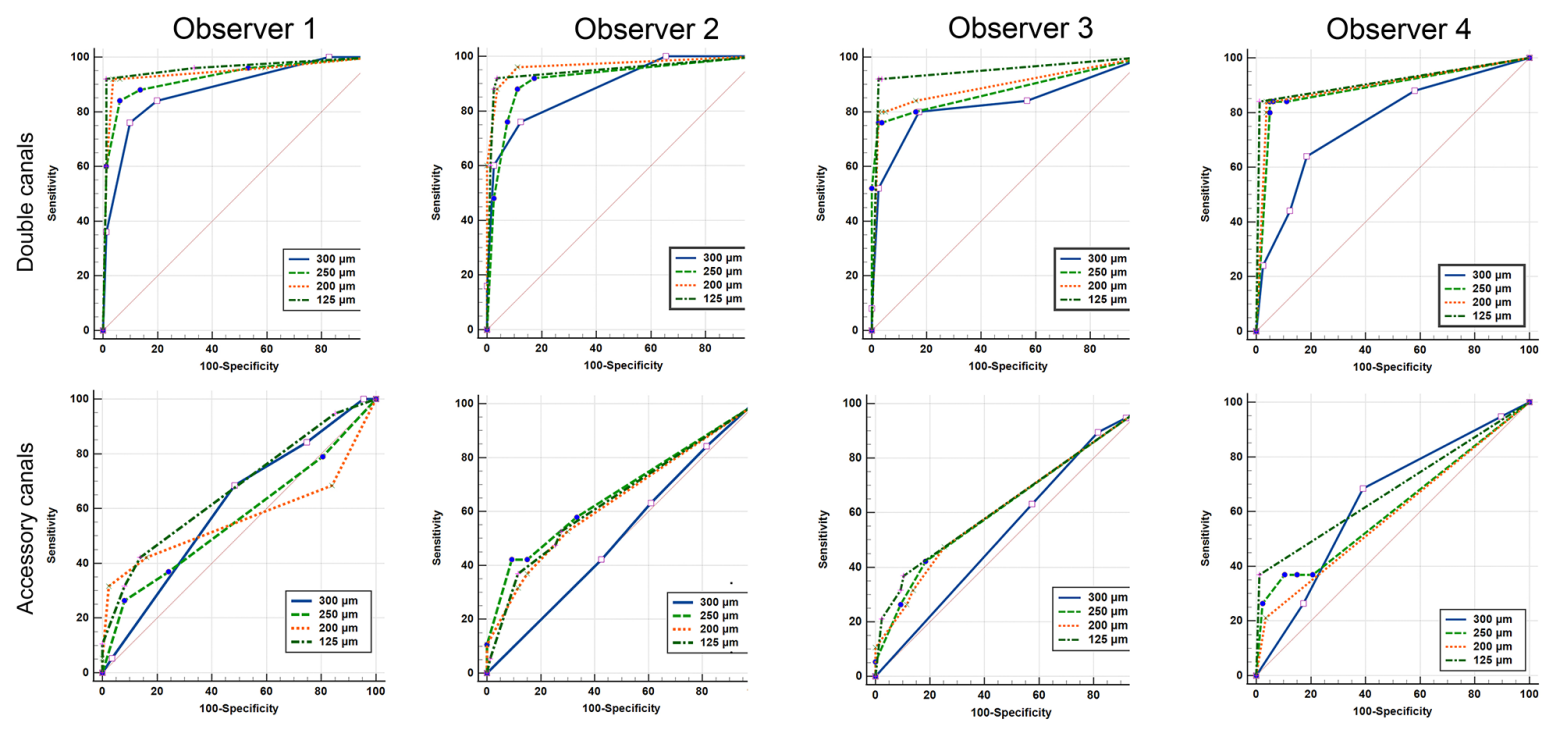
ROC curves for CBCT image sets taken at 4 different voxel sizes in the detection of double root canal systems and accessory canals in mandibular incisors (assessed for 4 observers)

The AUC values, sensitivity, specificity, PPV, NPV, and *Youden* index calculated for 4 observers and CBCT image sets with 4 different voxel sizes in the detection of double root canal systems were summarized in Table 2. The AUC values ranged from 0.765 to 0.889 for voxel size of 300 μm, from 0.877 to 0.926 for 250 μm voxel size, from 0.893 to 0.967 for 200 μm voxel size, and from 0.914 to 0.967 for 125 μm voxel size. In general, the AUC values, sensitivity, and specialty increased with the decrease in the voxel size. Statistically significantly higher AUC values were detected in 125 μm voxel size images as compared to the image sets with 300 μm voxel size (*p* = 0.038) and 250 μm voxel size (*p* = 0.044) for Observer 1. For Observer 2, significantly higher AUC values were detected in 200 μm voxel size images as compared to those with 300 μm voxel size (*p* = 0.024). For Observer 3, significantly higher AUC values were detected in 125 μm voxel size images as compared to CBCT images at 300 μm voxel size (*p* = 0.009), and for Observer 4, AUC values for images at 250, 200, and 125 μm voxel sizes were significantly higher than that for 300 μm voxel size, and the *p* value was 0.047, 0.027 and 0.015, respectively (Table 2; Fig. 4). Statistical differences were also detected in AUC values between Observer 1 and 4 (*p* = 0.043), 2 and 4 (*p* = 0.017) at 300 μm voxel size, and between Observer 2 and 4 (*p* = 0.043) at 200 μm voxel size.

**Table 2:** AUC values, Se, Sp, PPV, NPV, and *Youden* index for 4 observers in the diagnosis of double-canaled mandibular incisors based on CBCT imaging with 4 different voxel sizes

	Voxel size	*AUC*	*SE*	*P value*	*Asymptotic 95% confidence interval*		*Se*	*Sp*	*PPV*	*NPV*	*Youden* *index*
	(µm)				*Lower bound*	*Upper bound*					
Observer 1	300	0.882	0.039	< 0.0001	0.805	0.936	0.760	0.901	0.904	0.924	0.661
(*Tang Y*)	250	0.926	0.036	< 0.0001	0.859	0.968	0.840	0.938	0.808	0.950	0.778
	200	0.943	0.035	< 0.0001	0.881	0.979	0.920	0.963	0.885	0.975	0.883
	125	0.959	0.028	< 0.0001	0.901	0.988	0.920	0.988	0.958	0.976	0.908
Observer 2	300	0.889	0.035	< 0.0001	0.814	0.942	0.760	0.877	0.655	0.922	0.637
(*Gu Y*)	250	0.917	0.034	< 0.0001	0.847	0.961	0.880	0.889	0.710	0.960	0.769
	200	0.967	0.023	< 0.0001	0.912	0.992	0.960	0.889	0.727	0.986	0.849
	125	0.948	0.029	< 0.0001	0.887	0.982	0.920	0.963	0.885	0.975	0.883
Observer 3	300	0.827	0.058	< 0.0001	0.741	0.893	0.800	0.827	0.588	0.931	0.627
(*Wu Y*)	250	0.877	0.047	< 0.0001	0.799	0.933	0.760	0.975	0.905	0.929	0.735
	200	0.893	0.043	< 0.0001	0.818	0.945	0.800	0.975	0.909	0.940	0.775
	125	0.947	0.029	< 0.0001	0.886	0.981	0.920	0.975	0.920	0.975	0.895
Observer 4	300	0.765	0.056	< 0.0001	0.672	0.842	0.640	0.815	0.516	0.880	0.455
(*Pei F*)	250	0.889	0.042	< 0.0001	0.814	0.942	0.840	0.951	0.840	0.951	0.791
	200	0.899	0.040	< 0.0001	0.825	0.949	0.840	0.963	0.875	0.951	0.803
	125	0.914	0.038	< 0.0001	0.843	0.996	0.800	0.975	0.909	0.940	0.775

AUC, area under curve; CBCT, cone beam CT; NPV, negative predictive value; PPV, positive predictive value; Se, sensitivity; SE, standard error; Sp, specificity

### CBCT images at a voxel size of 125 μm have a weak diagnostic potential in the detection of ACs in mandibular incisors

As shown in Table 3; Fig. 4, among 4 observers, the AUC values ranged from 0.509 to 0.625 for 300 μm voxel size, from 0.562 to 0.667 for 250 μm voxel size, from 0.565 to 0.638 for 200 μm voxel size, and from 0.644 to 0.678 for 125 μm voxel size. These data also indicated that CBCT images had no (*p* > 0.05) or only low (AUC < 0.7) diagnostic potential in the detection of ACs in mandibular incisors. No statistically significant differences were found among different voxel sizes in the detection of ACs for each observer (all *p* > 0.05). In general, CBCT images at a voxel size of 125 μm had a weak diagnostic potential in the detection of ACs in mandibular incisors (for each of all 4 observers, *p* values were in a range between 0.001 and 0.034), and the sensitivity was at a low level ranged from 36.8 to 42.1%, while the specialty was at a high level ranged between 86.2% and 98.9%.

**Table 3:** AUC values, Se, Sp, PPV, NPV, and *Youden* index for 4 observers in the diagnosis of accessory canals in the mandibular incisors based on CBCT imaging with 4 different voxel sizes

	Voxel size	*AUC*	*SE*	*P value*	*Asymptotic 95% confidence interval*		*Se*	*Sp*	*PPV*	*NPV*	*Youden* *index*
	(µm)				*Lower bound*	*Upper bound*					
Observer 1	300	0.604	0.064	0.105	0.505	0.698	0.684	0.517	0.236	0.882	0.202
(Tang Y)	250	0.562	0.077	0.421	0.463	0.658	0.263	0.920	0.417	0.851	0.183
	200	0.565	0.091	0.473	0.466	0.661	0.316	0.977	0.750	0.867	0.293
	125	0.671	0.064	0.008	0.573	0.759	0.421	0.862	0.400	0.872	0.283
Observer 2	300	0.509	0.069	0.896	0.410	0.607	0.842	0.184	0.184	0.842	0.026
(Gu Y)	250	0.667	0.072	0.020	0.568	0.755	0.421	0.908	0.500	0.878	0.329
	200	0.638	0.069	0.047	0.526	0.729	0.526	0.701	0.278	0.871	0.228
	125	0.644	0.068	0.034	0.545	0.735	0.368	0.885	0.412	0.865	0.254
Observer 3	300	0.539	0.062	0.529	0.440	0.637	0.895	0.184	0.193	0.889	0.079
(Wu Y)	250	0.626	0.064	0.049	0.527	0.718	0.421	0.816	0.333	0.866	0.237
	200	0.633	0.068	0.051	0.534	0.725	0.368	0.874	0.389	0.864	0.242
	125	0.661	0.065	0.013	0.563	0.750	0.412	0.899	0.437	0.889	0.311
Observer 4	300	0.625	0.065	0.053	0.526	0.717	0.684	0.609	0.277	0.898	0.293
(Pei F)	250	0.577	0.067	0.251	0.477	0.672	0.263	0.977	0.714	0.859	0.240
	200	0.588	0.049	0.073	0.488	0.683	0.211	0.966	0.571	0.848	0.176
	125	0.678	0.057	0.001	0.581	0.766	0.368	0.989	0.875	0.878	0.357

AUC, the area under the curve; CBCT, cone beam CT; NPV, negative predictive value; PPV, positive predictive value; Se, sensitivity; SE, standard error; Sp, specificity

## Discussion

CBCT is a valuable diagnostic tool in endodontics. The selection of an appropriate scanning protocol is critically important to balance between maintenance of essential image quality and the reduction of radiation dose [30]. The voxel size is one of the most important scanning parameters, and in most of the cases, an optimal, rather than the minimum voxel size is desirable for achieving the best diagnostic efficiency, as a smaller voxel size is associated with more radiation exposure, increased noise level and decreased contrast-to-noise ratio, while might provide a same diagnostic outcome as lower resolution images [16, 21, 31, 32].

In the current ex vivo study, to identify the presence of a double root canal system and ACs in mandibular incisors, extracted teeth were fixed in the tooth sockets of dry mandibles with wax to simulate the in vivo condition. All observers assessed CBCT image sets taken at 4 different voxel sizes as our clinical routine, while the micro-CT images at 9 μm voxel size were used as the gold standard. During ROC curve analysis, a high AUC value closing 1.0 indicated an ideal diagnostic test, while a low AUC value closing 0.5 indicated no diagnostic effectiveness. The data in Table 3 reveals that all 4 voxel sizes are reliable in identifying the double root canal system (the AUC ranged from 0.765 to 0.967, all *p* < 0.0001), and for all 4 observers, a voxel size of 125 μm could achieve high diagnostic accuracy (all AUC > 0.9, and all *p* < 0.0001), while a voxel size of 300 μm could only achieve moderate diagnostic accuracy (the AUC ranged from 0.765 to 0.889). Professional experience is also an influencing factor, as for Observer 1 (a researcher with 9 years of working experience in radiology) and Observer 2 (an experienced endodontic specialist), the AUC values were greater than those of Observer 3 and 4 (both were graduate students of dental school) at each voxel size. Statistical differences were detected between Observer 1 and 4, and Observer 2 and 4 at 300 μm voxel size, and between Observer 2 and 4 at 200 μm voxel size (all *p* < 0.05). These findings also indicate that sharp CBCT images with high resolution (125 μm voxel size) are more essential for an inexperienced clinician; while for an experienced specialist, CBCT with a lower resolution (200 μm voxel size) is only slightly less accurate than the high-resolution modality (125 μm voxel size), which has an advantage of lower exposure dosage. This is in contrast to the report of Bauman et al. [22], who noted that the accuracy of MB2 canal detection couldn’t be correlated with the observers’ level of clinical experience. Previous scholars demonstrated that the presence of artifacts can also affect the diagnostic ability of CBCT [33]. Vizzotto et al. [23] found that the root-filling materials in MB1 may affect the detection of MB2, and the degree of influence was dependent on the CBCT voxel size during the examination. Recently, Lee et al. [34] retrospectively examined CBCT images of a Korean population and reported that the mandibular first molar showed a high incidence (27.0%) of separate disto-lingual (DL) roots, and this root variation was highly associated with the presence of two‑canaled mandibular incisors (left central incisors: *p* = 0.001, odds ratio = 4.25; left lateral incisors: *p* < 0.001, odds ratio = 3.8; right central incisors: *p* = 0.003, odds ratio = 3.86; right lateral incisors: *p* = 0.001, odds ratio = 3.44). Similar finding has been reported in the Taiwanese population [10, 11], which indicates that when the voxel size and quality of CBCT images are inadequate for accurate detection of two-canaled mandibular incisors, the root number of the mandibular first molars may provide clinicians some clues. The DL root can more easily be diagnosed, and its presence indicates a high possibility of two canals in mandibular incisors on the same side.

Literature review shows only very few scholars have previously assessed the diagnostic potential of CBCT in identifying ACs [16, 17]. To evaluate the effectiveness of CBCT imaging (using a voxel size of 80 μm) for the detection of lateral canals in endodontically treated premolars, a similar ex vivo study was carried out by Sousa et al. [16]. They reported that the AUC value was only 0.58 and 0.49 (all *p* < 0.001) before and after root canal treatment, respectively, and CBCT imaging was not an effective diagnostic tool for the detection of ACs. The current study found that CBCT images taken at 125 μm voxel size have a low (AUC: 0.5–0.7) diagnostic potential in the detection of ACs in mandibular incisors for all 4 observers. The AUC values ranged from 0.638 to 0.678 (all *p* < 0.05), and the sensitivity was at a low level ranging between 36.8% and 57.9%, while the specialty was at a relatively high level ranging between 73.6% and 98.8%. The higher specificity value can be due to the observers’ difficulty in detecting the accessory canals and they tended to score “4 = probably absent” or “5 = absent”, which resulted in a large number of “negative” answers, consequently increasing the number of “true-negative” cases [16]. This finding is similar to that of Ji et al. [17], who reported that partial lateral accessory canals could be detected using CBCT at high-resolution scan mode (voxel size = 125 μm). Theoretically, CBCT cannot identify an anatomic structure with a size below the voxel size. Moreover, the relationship between CBCT spatial resolution and voxel size indicated that the former is at least twice greater than the latter [35]. The odontometric measurement by micro-CT showed that the majority of the ACs had a canal size below 250 μm (Fig. 3), which means even using the smallest voxel size of 125 μm, the spatial resolution (which is often 2-fold greater than the voxel size) is inadequate to identify the majority of ACs. The low sensitivity also indicated that the majority of ACs were missed diagnoses. Theoretically, more advanced modulates with 75–80 μm voxel size can improve diagnostic accuracy.

This ex vivo study has several limitations. First, to simulate the in vivo dental anatomy, the extracted sample teeth were fixed in the tooth sockets of dry mandibles, which were stationary during CBCT scanning. While in the clinical scenario, patient movement is also an influential factor, with the magnitudes even exceeding voxel size and scatter effects, and this may lead to overestimated diagnostic accuracy of the CBCT. Second, in the current study, the majority of the sample teeth were collected from elderly people, and the small size of the main canals or ACs was associated with a lower CBCT detection rate. Finally, this study only used one CBCT machine (3D eXam I, KaVo) with the smallest voxel size of 125 μm, which was not the most advanced machine currently in the CBCT market. Therefore, further studies are essential based on other brands of CBCT units, especially those with a minimum voxel size of 75–80 μm.

## Conclusions

CBCT with 300 μm voxel size could only provide moderate diagnostic accuracy in the detection of double canal systems in mandibular incisors. CBCT with a voxel size of 125 μm exhibited high diagnostic value in the detection of double canal systems while showing low but statistically significant value in the detection of ACs. For experienced observers, lower resolution CBCT at 200 μm voxel size only slightly reduces the diagnostic accuracy, while having the advantage of radiation reduction.

## Data Availability

All the datasets used and analyzed during the current study are available from the corresponding author on reasonable request.

## Declarations

### Abbreviations

CBCT Cone-beam computed tomography.

micro-CT micro-computed tomography.

3D three-dimensional.

FOV field of view.

AC accessory canal.

ROC receiver operating characteristic.

AUC area under curve.

MB2 secondary mesio-buccal canal.

MB1 first mesio-buccal canal.

DL disto-lingual.

### Authors details

^1^The Stomatology Hospital Affiliated of Suzhou Vocational Health College, Renmin Road 829#, Gusu Dist., 215002 Suzhou, China.^2^ Department of Central Laboratory and Dentistry, Ninth People’s Hospital of Suzhou, Soochow University, Ludang Road 2666#, Wujiang Dist., 215200 Suzhou, China.
